# Liver AMP-Activated Protein Kinase Is Unnecessary for Gluconeogenesis but Protects Energy State during Nutrient Deprivation

**DOI:** 10.1371/journal.pone.0170382

**Published:** 2017-01-20

**Authors:** Clinton M. Hasenour, D. Emerson Ridley, Freyja D. James, Curtis C. Hughey, E. Patrick Donahue, Benoit Viollet, Marc Foretz, Jamey D. Young, David H. Wasserman

**Affiliations:** 1 Department of Molecular Physiology and Biophysics, Vanderbilt University, Nashville, Tennessee, United States of America; 2 Mouse Metabolic Phenotyping Center, Vanderbilt University, Nashville, Tennessee, United States of America; 3 Inserm, U1016, Institut Cochin, Paris, France; 4 CNRS, UMR 8104, Paris, France; 5 Université Paris Descartes, Sorborne Paris Cité, Paris, France; 6 Department of Chemical and Biomolecular Engineering, Vanderbilt University, Nashville, Tennessee, United States of America; Université catholique de Louvain, BELGIUM

## Abstract

AMPK is an energy sensor that protects cellular energy state by attenuating anabolic and promoting catabolic processes. AMPK signaling is purported to regulate hepatic gluconeogenesis and substrate oxidation; coordination of these processes is vital during nutrient deprivation or pathogenic during overnutrition. Here we directly test hepatic AMPK function in regulating metabolic fluxes that converge to produce glucose and energy *in vivo*. Flux analysis was applied in mice with a liver-specific deletion of AMPK (L-KO) or floxed control littermates to assess rates of hepatic glucose producing and citric acid cycle (CAC) fluxes. Fluxes were assessed in *short* and *long* term fasted mice; the latter condition is a nutrient stressor that increases liver AMP/ATP. The flux circuit connecting anaplerosis with gluconeogenesis from the CAC was unaffected by hepatic AMPK deletion in *short* and *long* term fasting. Nevertheless, depletion of hepatic ATP was exacerbated in L-KO mice, corresponding to a relative elevation in citrate synthase flux and accumulation of branched-chain amino acid-related metabolites. L-KO mice also had a physiological reduction in flux from glycogen to G6P. These results demonstrate AMPK is unnecessary for maintaining gluconeogenic flux from the CAC yet is critical for stabilizing liver energy state during nutrient deprivation.

## Introduction

Fasting [1] and exercise [2, 3] provoke a reciprocal rise and fall in hepatic AMP and ATP concentrations. AMP-activated protein kinase (AMPK) monitors fluctuations in adenine nucleotide ratios (AMP/ATP and ADP/ATP) and directs signaling pathways that control nutrient flux [4]. Targets of AMPK regulation are involved in the acute and chronic control of numerous cell processes, including lipid [5–9], protein [10, 11], glucose [5, 12–15], and energy metabolism [16–18]. Moreover, AMPK is critical for the maintenance of hepatic energy homeostasis during pharmacological energy stress [17, 19].

AMPK activation is observable in conditions when glucagon action is high [1]. Glucagon stimulates gluconeogenesis by increasing hepatic fat oxidation [20], amino acid extraction [21], and intrahepatic conversion of precursors to glucose [22]. Tracer studies *in vivo* and in perfused liver have substantiated the tight relationship between hepatic oxidative metabolism, energy production, and gluconeogenesis [23–27]. The increase in AMPK activity during glucagon stimulation suggests that its main physiological role may be to sustain oxidative metabolism to support—rather than inhibit—gluconeogenesis. This hypothesis contrasts with findings that suggest AMPK is an inhibitor of gluconeogenesis.

The present studies examine the role of hepatic AMPK in the metabolic response of the liver to a physiological reduction in ATP during the nutrient deprivation of progressive fasting. This was accomplished by combining comprehensive liver metabolomics with *in vivo* metabolic flux analysis (MFA). Liver-specific AMPKα1α2 knockout (L-KO) and control (WT) mice were used to evaluate AMPK’s role in fluxes linking energy metabolism with glucose production. Specifically, anaplerotic, cataplerotic, and citric acid cycle (CAC) fluxes were measured along with the conversion of phosphoenolpyruvate, glycerol, and glycogen to glucose in conscious, unrestrained mice. The application of MFA in this context provides a dynamic readout of AMPK function in regulating hepatic intermediary metabolism *in vivo*. Hepatic AMPK deletion exacerbates the decline in liver ATP. This corresponds to abnormal CAC and branched-chain amino acid/keto acid (BCAA/BCKA)-related metabolism. Despite these effects, liver AMPK deletion has no impact on *in vivo* gluconeogenic flux from the CAC with progressive fasting.

## Materials and Methods

### Animal models

All procedures were approved by Vanderbilt University Animal Care and Use Committee. All mice used in this study were bred in the Vanderbilt University Division of Animal Care. To generate liver-specific AMPK knockout mice, Alfp-*Cre*+ mice were crossed with mice containing floxed AMPK α1 and α2 subunits on a C57BL/6 background. Genotyping or western blots were performed to confirm liver-specific deletion of AMPK—defined here as L-KO or floxed control (WT) mice. Mice were maintained on a 12:12 hr light/dark cycle in a temperature- and humidity-controlled environment. At 3 wks, mice were weaned on a standard chow diet (5001 Purina Laboratory Diet) with free access to water.

### Surgical procedures and stable-isotopic infusions

Catheters were implanted in the left common carotid artery and right jugular vein in 14 wk-old, male WT and L-KO mice as previously described [28]. Mice were individually housed post-surgery and those that returned to ≥85% of their pre-surgical weight were studied. Experiments were performed 5–9 days post-surgery. During catheterization, mice received inhaled isofluorane as a general anesthesia (2% at induction and to maintain) and subcutaneous ketofen as an analagesia (2 mg/ml during surgery and as needed).

Food was withdrawn at the start of the light cycle (06:00hrs) for studies in *short* term fasted (~9 hr) mice. A ^2^H_2_O (99.9%)-saline bolus was infused intravenously 3.5 h into the fast over a 25min period to enrich total body water to 4.5% [29]. An 80μL arterial sample was drawn prior to the ^2^H_2_O bolus to measure natural isotopic enrichment of glucose. A [6, 6-^2^H_2_]glucose (99%) prime (440 μmol•kg^-1^) was dissolved in the ^2^H_2_O bolus. A separate, continuous infusion of [6, 6-^2^H_2_]glucose (4.4 μmol•kg^-1^•min^-1^) began following the bolus. A primed (1.1 mmol•kg^-1^), continuous (0.055mmol•kg^-1^•min^-1^) infusion of [^13^C_3_]propionate (99%, sodium salt) was administered 3.5 hrs after the ^2^H_2_O bolus and [6, 6-^2^H_2_]glucose prime [29]. Three arterial samples were taken in the isotopic steady state (90–110 min following the [^13^C_3_]propionate bolus) to determine the mass isotopomer distribution (MID) of plasma glucose for metabolic flux analysis (MFA). A similar set of plasma samples was obtained prior to [^13^C_3_]propionate delivery. Studies in *long* term (~20hr) fasted mice were performed identically to those of *short* term fasted mice except fasting commenced at the start of the dark cycle and the ^2^H_2_O bolus was administered 14.5 hrs later. Donor erythrocytes were infused throughout the study to prevent a drop in hematocrit. Stable isotopes were purchased from Cambridge Isotope Laboratories, Inc (Tewksbury, MA, USA). All infusates were prepared in 4.5% ^2^H_2_O enriched saline unless otherwise noted. Arterial blood glucose levels were monitored using an AccuCheck Glucometer (Roche Diagnostics, Indianapolis, IN, USA).

### Metabolic Flux Analysis (MFA)

**Fig 1:**
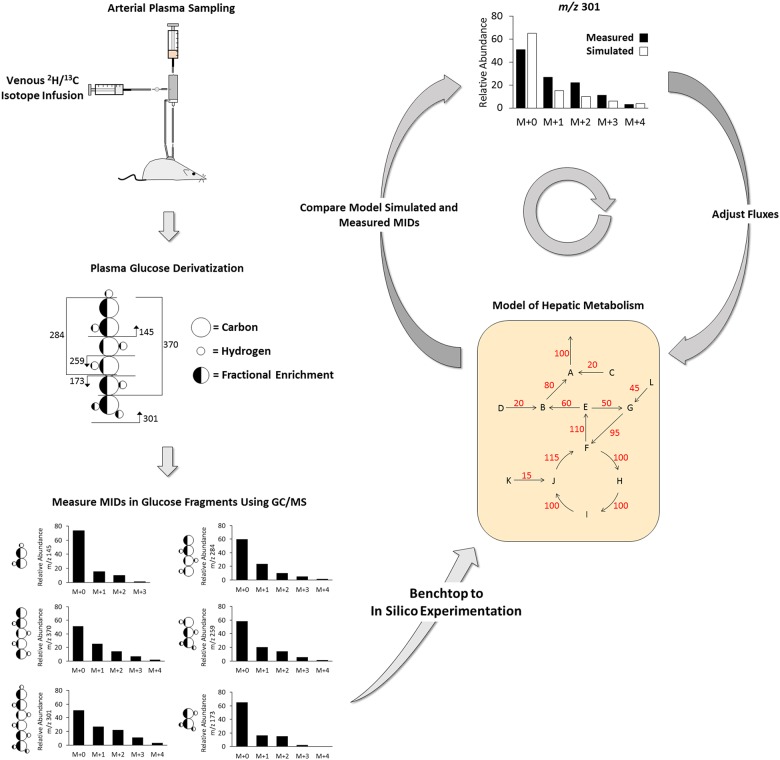
Measurement of hepatic glucose and oxidative metabolism *in vivo*. The workflow depicted in Fig 1 transitions from *in vivo* isotope delivery and plasma sampling to *in silico* flux analysis. ^2^H and ^13^C isotopes delivered intravenously in consciously catheterized mice enrich glucose produced from the liver. Plasma glucose samples obtained during the isotopic steady-state are derivatized and analyzed through GC-MS analysis [30]. Glucose-fragment MIDs—m/z 173–176, 259–263, 284–287, 370–374, 145–147, and 301–311—are integrated from MS peaks. A previously generated model of hepatic metabolism is used to simulate glucose fragments MIDs using INCA (custom MFA software) [29]. Flux estimates are regressed by minimizing the difference between simulated and empirically measured MIDs.

A complete description of the metabolic flux methodology used in these studies is detailed elsewhere [29]. Briefly, an *in vivo*, stable-isotopic GC-MS based microassay was used to measure relative and absolute rates of glucose and CAC-related fluxes. The workflow for performing MFA of *in vivo* hepatic glucose and oxidative metabolism is illustrated in Fig 1. The schematic summarizes the experimental and analytical protocol, beginning at isotope delivery in conscious, unrestrained mice and ending at model-based regression of fluxes.

Three 40 μL arterial plasma samples were obtained during the isotopic steady state; plasma samples were divided into 3 aliquots, deproteinized, and derivatized into di-*O*-isopropylidine propionate, aldonitrile pentapropionate, and methyloxime pentapropionate glucose derivatives, as described by Antoniewicz et al [30]. These glucose derivatives were dissolved in ethyl acetate, transferred to GC injection vials, and analyzed in splitless mode using an Agilent 7890A gas chromatography system equipped with an HP-5s capillary column interfaced with an Agilent 5975C mass spectrometer. Mass spectrometry data were collected in scan mode from *m/z* 300–320 for di-*O*-isopropylidine derivatives, *m/z* 100–500 for aldonitrile derivatives, and *m/z* 144–260 for methyloxime derivatives. Peaks were integrated in MATLAB to obtain mass isotopomer distributions (MIDs) for six ion ranges: aldonitrile, *m/z* 173–176, 259–263, 284–287, 370–374; methyloxime, *m/z* 145–147; di-*O*-isopropylidine, *m/z* 301–311. MIDs for glucose derivative fragments are depicted in Fig 1 and used to regress fluxes from a molecular model of hepatic metabolism.

**Fig 2:**
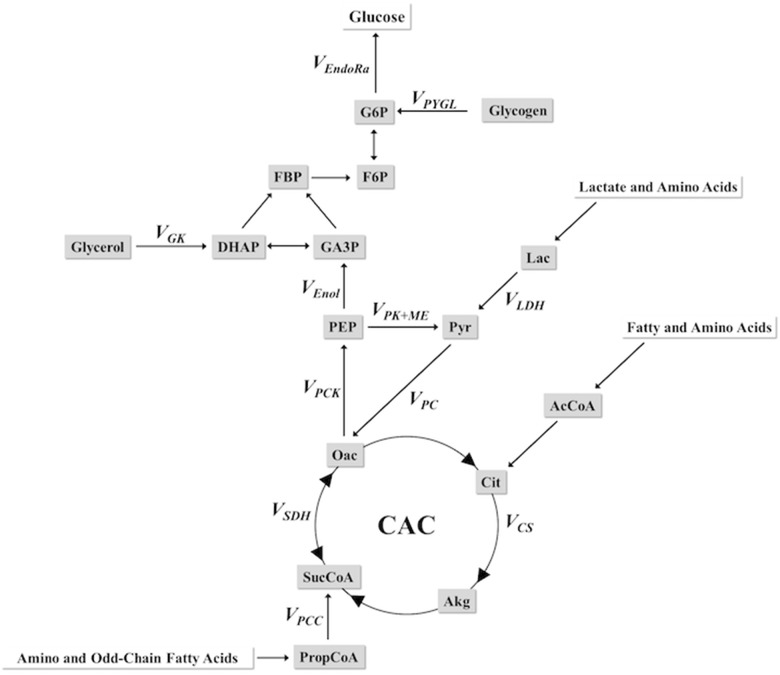
Scheme of glucose producing and CAC-related fluxes for MFA. Metabolites modeled for intermediary metabolic exchange include lactate, glycerol, glycogen, amino and fatty acids. SucCoA serves as the entry point for [^13^C_3_]propionate into the CAC and ^2^H from ^2^H_2_O enters at multiple loci in the flux model. A complete description of the molecular reaction network, assumptions, limitations, and flux analysis used in these studies has been elaborated on elsewhere (31). Multiple substrates shuttle through Pyr to the CAC and, thus, ***V***_***LDH***_ encompasses all non-PEP derived, unlabeled sources of anaplerotic flux. Total anaplerotic flux is the sum of anaplerotic inputs (***V***_***PC***_ + ***V***_***PCC***_) and is equal to cataplerosis (***V***_***PCK***_). Abbreviations and a description of metabolites and central reactions in the flux model are listed in the **Abbreviations** subsection.

A molecular reaction network [29] was constructed using the INCA software package [31] (accessible at http://mfa.vueinnovations.com/mfa) describing the biochemical reactions linking hepatic glucose and CAC-metabolism. The complete model, available in the Appendix of Hasenour et al. [29], has been simplified into the scheme in Fig 2. The assumptions of the model are described elsewhere with a discussion of the strengths and limitations of the approach [23–26, 29]. Hydrogen and carbon atom transitions were defined for each reaction; the integration of ^2^H and ^13^C from ^2^H_2_O, [6, 6-^2^H_2_]glucose, and [^13^C_3_]propionate were introduced through specific loci in the network. Model-based regression of fluxes was performed using the molecular network to track carbon and hydrogen atoms through the network. The model consists of 19 biochemical reactions, 22 metabolic nodes, and 424 mass isotopomer balance equations. Flux through each reaction was estimated relative to ***V***_***CS***_ (fixed at 100) by minimizing the sum of squared residuals between simulated and experimentally determined MIDs. Flux estimates were repeated 25 times from random initial values. A chi-square test was used to assess goodness-of-fit. Confidence intervals of 95% were computed by evaluating the sensitivity of the sum of squared residuals to variations in flux values [32]. All fits were accepted based on a chi-square test (*p* = *0*.*05*) with 22 degrees of freedom (i.e., the regressions were overdetermined by 22 measurements). The [6, 6-^2^H_2_]glucose (***V***_***Inf***_) infusion rate and mouse weights were used to convert relative fluxes to absolute rates. Flux estimates for the steady state samples were averaged to obtain rates for each mouse.

The [^13^C] isotope used in this study has been independently tested by correlating TCA cycle flux to oxygen consumption in perfused liver, by measuring *in vivo* glucose production in the presence and absence of propionate, and by direct comparison of propionate and lactate isotopes [33]. Intravenous [^13^C_3_]propionate at the dose used here does not result in detectable increases in glucose production [29]. Furthermore, relative flux estimates of hepatic glucose and oxidative metabolism fall within ranges observed by other groups using various isotopes that enter hepatic metabolism through anaplerotic reactions [25, 34–37]. Tracer applications similar to those used here have been critically reviewed recently [38]. It is notable that all stable isotope approaches involve limitations and assumptions, which are often inherent to the tracer selected. Isotope selection for measuring hepatic oxidative and glucose metabolism has recently focused on the measurement of pyruvate cycling flux *V*_*PK+ME*_ [33, 39]. Removal of *V*_*PK+ME*_ from the model based-regression in the present study increases the SSR in short and long-term fasted C57Bl/6J mice. in the latter, the SSR exceeds the threshold for an acceptable fit. The importance of this parameter for fitting isotopomer data to models of hepatic metabolism is underscored by historical and more recent literature. Several groups over the last few decades have detected a quantitatively important role for pyruvate cycling in hepatic metabolism under certain conditions. This is consistent for groups using a variety of radio- and stable isotopic tracers and modeling approaches [24, 25, 27, 29, 34, 35, 37, 40, 41]. Relative to anaplerosis, the estimates of *V*_*PK+ME*_ reported in the present manuscript are within the range measured in rodents *in vivo* and liver perfusion across several previous studies.

### Immunoblotting

A separate cohort of uncatheterized, 14wk-old, *short* (~7hr) and *long* (~18hr) term fasted WT and L-KO mice were sacrificed for molecular signaling, lipid, and metabolite analysis. Mice were euthanized through cervical dislocation and liver tissue was rapidly excised and submerged in liquid nitrogen to limit potential changes in metabolites. Liver was homogenized in an extraction buffer (1 mg/10 μl) (50 mM Tris; 1 mM EDTA; 1 mM EGTA; 10% glycerol; 1% Triton X-100, pH 7.5) containing protease and phosphatase inhibitors. Protein isolates were denatured and reduced at 70°C and separated in NuPAGE 4–12% (v/v) Bis-Tris (Invitrogen, Carlsbad, CA, USA) or 7.5% Mini-PROTEAN (Bio-Rad, Hercules, CA, USA) gels and transferred to a PVDF membrane. Membranes were probed for tAMPK, pAMPK^T172^, pACC^S79^, tACC, tAkt, and pAkt^S473^ (Cell Signaling Technology, Danvers, MA, USA) and HRP-linked α-rabbit secondary antibodies were applied for ECL and visualization. ImageJ software was used for densitometry.

### Liver lipid, nucleotide, glycogen, and metabolomics analysis

Liver lipids were isolated through Folch extraction [42]. Hepatic adenine nucleotides were measured as described elsewhere [2]. Briefly, liver tissue was rapidly homogenized in 0.4 M HClO_4_-0.5 mM EGTA (1 mg/10 μL), spun down and neutralized with 0.5 M K_2_CO_3_. Samples were centrifuged again and the supernatants were saved for HPLC analysis. Energy charge (EC) was calculated with the following equation: EC = ([ATP] + 0.5[ADP])/([ATP] + [ADP] + [AMP]). Liver glycogen was determined from tissue extracts [43]. Freeze-clamped liver tissue (0.05–0.1 g) was sent to Metabolon for metabolite and statistical analysis [44]. Briefly, liver protein was precipitated through a methanol extraction and centrifuged. The supernatant was separated and dried for further preparation for LC+, LC-, and GC analysis. For LC/MS/MS analysis, samples were reconstituted in basic or acidic LC-compatible solvents, loaded onto columns (Waters UPLC BEH C18) and gradient-eluted with water and methanol containing either formic acid or ammonium bicarbonate. Dried samples designated for GC/MS were derivatized using BSTFA. Samples were analyzed with a GC column composed of 5% phenyldimethylsilicone and mass spectrometry was performed with a Thermo-Finnigan Trace DSQ fast-scanning single-quadrupole mass spectrometer using electron impact ionization. Metabolites were identified by a comparison to library entries of purified standards. Proprietary visualization and interpretation software were used to match chromatographic properties/mass spectra with specific compounds. ANOVA contrasts and Two-Way ANOVAs (p≤0.05) were used to determine whether two means of a population were different.

### Statistical analysis

Two-tailed *t* tests, Two-Way ANOVAs and Tukey post-hoc tests were used to determine specific differences, unless otherwise indicated. Data are expressed as means ± SEM. Significance was *p≤0*.*05*.

## Results

### AMPK attenuates fasting-mediated changes in glucose and CAC-flux but not gluconeogenesis

**Fig 3:**
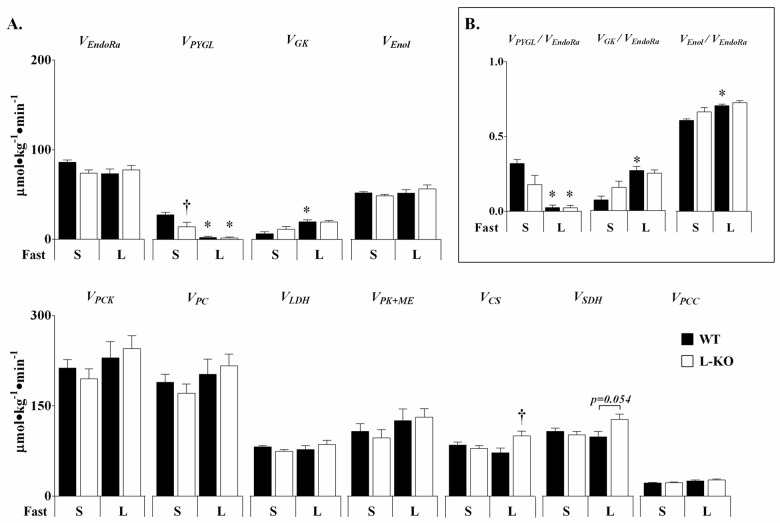
Abnormal glucose and oxidative fluxes in mice lacking hepatic AMPK. Absolute fluxes (μmol•kg^-1^•min^-1^) (A) were determined for *short* (**S**) and *long* (**L**) term fasted WT and L-KO mice. Relative contributors to ***V***_***EndoRa***_ (B) were determined by dividing ***V***_***PYGL***_, ***V***_***GK***_, and ***V***_***Enol***_ by ***V***_***EndoRa***_; ***V***_***GK***_ and ***V***_***Enol***_ are presented in hexose units such that ***V***_***PYGL***_+***V***_***GK***_+***V***_***Enol***_ = ***V***_***EndoRa***_. Data are presented as means ± SEM, *n = 5–7* in each group. *p≤0.*05* vs. *short* term fasting; †*p≤0*.*05* vs. WT mice.

The transition from a *short* to *long* term fast resulted in established changes in glucose metabolism in WT mice (Fig 3A and 3B). Glucose production (***V***_***EndoRa***_) trended toward a reduction with *long* term fasting (Fig 3A). The absolute rate of glucose flux from glycogen (***V***_***PYGL***_) reduced to negligible rates while that from glycerol (***V***_***GK***_) increased with fast duration (Fig 3A and 3B). The reduction in glycogenolytic glucose production between *short* and *long* term fasting corresponded to a large reduction in liver glycogen (27.8±3.2 to 6.6 ±0.8mg/gLiver). Glucose production in *long* term fasted WT mice was almost entirely gluconeogenic, emanating predominantly from PEP (Fig 3A and 3B). Glucose and CAC-related fluxes in *long* term fasted WT mice exhibited similar characteristics to those observed in the same strain previously [29]. Furthermore, absolute flux rates in *long* term fasted mice measured here are comparable to measurements made using NMR methods in overnight fasted mice [37].

*Short* term fasted L-KO mice exhibited qualitative and quantitative characteristics of a prolonged fasted mouse. ***V***_***EndoRa***_ in L-KO mice trended lower than *short* term fasted WT mice, stemming from a ~50% reduction in glucose flux from glycogen (***V***_***PYGL***_) (Fig 3A and 3B). The reduction in ***V***_***PYGL***_ corresponded to a shift toward increased reliance on gluconeogenesis (Fig 3A and 3B). Though glucose flux from glycerol (***V***_***GK***_) was not significantly different than controls (Fig 3A and 3B), intrahepatic glycerol levels were elevated in *short* term fasted L-KO mice (Table 1). The rates of glucose production and its associated sources (Fig 3A and 3B) in WT and L-KO mice converged with a long fast, as L-KO mice experienced a reduction in liver glycogen (22.1±3.7 to 6.5 ±1.2mg/g Liver) similar to that observed in WT-mice.

**Table 1:** Metabolites of the CAC and glucose producing pathways.

	Effect of Fasting		Effect of Genotype	
*Metabolites of the CAC and Glucose Producing Pathways*	WT Long WT Short	L-KO Long L-KO Short	L-KO Short WT Short	L-KO Long WT Long
glycerol	1.84*	1.46*	1.31*	1.04
3-phosphoglycerate	1.3*	0.88	1.64*	1.11
pyruvate	0.82	0.70*	1.39*	1.19
lactate	0.36*	0.45*	1.16	1.46*
citrate	0.56*	0.59*	0.83	0.88
fumarate	0.48*	0.57*	0.95	1.12
malate	0.55*	0.64*	0.97	1.12

Liver metabolites in glucose production and the CAC were determined through metabolomics analysis of *short* and *long* term fasted WT and L-KO mice. CAC-related metabolites were included in the table. Data are expressed as ratios, *n = 7–8* in each group.

*represents *p*≤0.05 between identified groups.

Hepatic AMPK deletion significantly affected CAC flux in *long* term fasted mice (Fig 3A). Specifically, ***V***_***CS***_ flux was elevated in *long* term fasted L-KO mice. However, no differences in gluconeogenesis from the CAC (***V***_***Enol***_), anaplerosis (***V***_***PC***_), cataplerosis (***V***_***PCK***_), or pyruvate cycling (***V***_***PK+ME***_) were observed (Fig 3A). CAC intermediates displayed similar responses to fasting in WT and L-KO mice (Table 1).

### AMPK is critical for maintaining hepatic energy homeostasis in long term fasting

**Fig 4:**
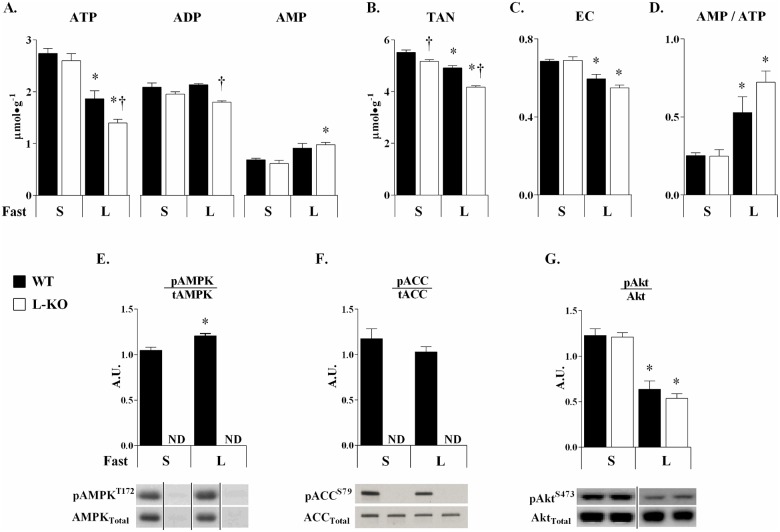
AMPK protects against fasting-mediated deficits in liver ATP. Hepatic adenine nucleotides (A), the total adenine nucleotide pool (TAN = ATP + ADP + AMP) (B), energy charge (EC = [ATP + 0.5ADP]/[TAN]) (**C**.), and the AMP/ATP ratio (D) were determined for WT and L-KO mice in *short* (**S**) and *long* (**L**) term fasting. Liver AMPK (E and F) and Akt (G) signaling in *short* and *long* term fasted mice. Phosphorylated to total AMPK, ACC, Akt are provided as ratios (A.U.). Black lines separating lanes denote images obtained either from portions of the same or separate blots. Data are expressed as means ± SEM, *n = 6–7* in each group. *p≤0.*05* vs. *short* term fasting; †*p≤0*.*05* vs. WT mice.

Long term fasting reduced hepatic ATP and energy charge while increasing the AMP/ATP ratio in WT mice (Fig 4A, 4C and 4D). AMP-deaminases work to clear elevations in AMP and, in effect, may reduce the adenine nucleotide pool. *Long* term fasting reduced TAN (Fig 4B). The increase in the AMP/ATP ratio also corresponded to a modest increase in AMPK activation (pAMPK^T172^/AMPK) (Fig 4E). Phosphorylation of a downstream AMPK target, acetyl-CoA carboxylase (pACC^S79^/ACC), was not statistically different from that of *short* term fasted WT mice (Fig 4F). A decrease in Akt (pAkt^S473^/tAkt) phosphorylation was also observed in the *long* term fast (Fig 4G).

The absence of hepatic AMPK resulted in a greater decline in hepatic energy state in *long* term fasting. Indeed, ATP and ADP levels were significantly lower in *long* term fasted L-KO mice compared to WTs (Fig 4A). TAN levels were reduced in *short* term fasted L-KO mice, which was exacerbated by extending fast duration (Fig 4B). These data suggest that AMPK-deficient livers may rely on greater AMP degradation to limit the rise in AMP/ATP and preserve energy charge (Fig 4C and 4D). The larger, fasting-mediated decrease in hepatic ATP corresponded to a relative elevation of ***V***_***CS***_ in L-KO mice (Fig 3A). The acceleration in ***V***_***CS***_ may be compensatory as mitochondria isolated from AMPK deficient livers have impaired mitochondrial efficiency [17]. AMPK indirectly supports mitochondrial fatty acid (FA) transport and, likely, β-oxidation [6]. Impairments in the generation and/or utilization of reducing equivalents from FAs in the liver may contribute to abnormal energy metabolism observed in the absence of hepatic AMPK during *long* term fasting.

### An early elevation in fatty acids precedes normal triglyceride accumulation in liver AMPK knockout mice

**Fig 5:**
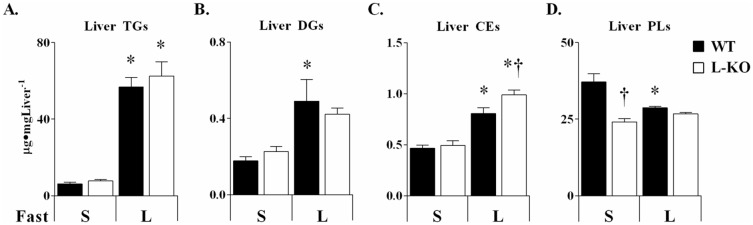
Liver AMPK-dependent and independent effects of fast duration on liver lipids. Liver triglycerides (TGs) (A), diglycerides (DGs) (B), cholesterol esters (CEs) (C), and phospholipids (PLs) (D) in WT and L-KO mice following a *short* (**S**) and *long* (**L**) term fast. Data are expressed as means ± SEM, *n = 6–7* in each group. *p≤0.*05* vs. *short* term fasting; †*p≤0*.*05* vs. WT mice.

Prolonged fasting promotes adipose tissue lipolysis through a decrease in circulating insulin and increases in glucocorticoids and catecholamines [45]. FAs sequestered by the liver undergo oxidation or re-esterification. Increasing fast duration elevated hepatic triglycerides (TGs), diglycerides (DGs), and cholesterol esters (CEs) (Fig 5A, 5B and 5C). Increases in these lipids corresponded to an elevation in several long and medium chain fatty acids in the livers of WT and L-KO mice (Table 2). In contrast, WT mice experienced a reduction in liver phospholipids (PL) which did not occur in the absence of hepatic AMPK (Fig 5D).

**Table 2:** Hepatic long-chain fatty acids, linoleic and arachidonic acid derivatives are elevated in AMPK-deficient livers of *short* term fasted mice.

	Effect of Fasting		Effect of Genotype	
*Long Chain Fatty Acids*	WT Long WT Short	L-KO Long L-KO Short	L-KO Short WT Short	L-KO Long WT Long
linoleate (18:2n6)	1.75*	1.53*	1.12*	0.97
linolenate (18:3n3 or 6)]	2.62*	2.14*	1.27*	1.04
eicosapentaenoate (EPA; 20:5n3)	1.54*	1.43*	0.91	0.85
myristate (14:0)	2.44*	2.09*	1.28*	1.10
myristoleate (14:1n5)	5.19*	4.18*	1.21	0.97
pentadecanoate (15:0)	2.04*	1.61*	1.30*	1.02
palmitate (16:0)	1.44*	1.30*	1.14*	1.02
palmitoleate (16:1n7)	1.76*	1.36	1.41	1.09
margarate (17:0)	1.76*	1.57*	1.13	1.01
10-heptadecenoate (17:1n7)	1.51*	1.29*	1.30*	1.10
stearate (18:0)	1.31*	1.14	1.08	0.94
oleate (18:1n9)	2.25*	1.74*	1.32*	1.02
cis-vaccenate (18:1n7)	1.61*	1.32*	1.25	1.03
stearidonate (18:4n3)	4.42*	3.17*	1.22	0.88
10-nonadecenoate (19:1n9)	1.56*	1.40*	1.09	0.97
arachidate (20:0)	0.71*	0.51*	1.54*	1.12
arachidonate (20:4n6)	1.20*	1.26*	1.01	1.05
erucate (22:1n9)	0.37*	0.30*	1.45*	1.20
docosadienoate (22:2n6)	0.54*	0.40*	1.50*	1.10
docosatrienoate (22:3n3)	0.55*	0.71	0.77	0.99
*Medium Chain Fatty Acids*				
caproate (6:0)	1.05	0.9	1.14	0.97
heptanoate (7:0)	0.71	0.78	1.07	1.17
caprylate (8:0)	1.14	0.98	1.09	0.94
laurate (12:0)	3.59*	3.41*	1.02	0.97
5-dodecenoate (12:1n7)	5.64*	4.99*	1.13	1
*Arachidonic and Linoleic Acid Derivatives*				
13-HODE + 9-HODE	1.95*	1.45*	1.23*	0.91
12-HETE	1.55*	1.08	1.3*	0.9
15-HETE	1.31*	1.04	1.16*	0.93

Liver long-chain fatty acid species, 9-HODE + 13-HODE, 12-HETE, and 15-HETE, and medium chain fatty acids were determined for *short* and *long* term fasted WT and L-KO mice through metabolomics analysis. Data are expressed as ratios, *n = 7–8* in each group.

*represents *p*≤0.05 between identified groups.

Liver TGs, DGs, and CEs (Fig 5A, 5B and 5C) were not different in *short* term fasting. Several long-chain FA species were, however, elevated in L-KO mice (Table 2). The increase in long-chain liver FAs occurred in both saturated and unsaturated species. Furthermore, the elevation in hepatic FAs corresponded to an increase in lipid derivatives of linoleic and arachidonic acid (Table 2, 9, 13-HODE and 12, 15-HETEs) [46]. In contrast, no genotype-specific differences were observed in medium-chain FAs (Table 2) providing further evidence for the importance of AMPK specifically in long-chain FA trafficking. Additionally, liver phospholipids were significantly reduced in *short* term fasted L-KO mice (Fig 5D). With the exception of CEs, which were elevated in *long* term fasted L-KO mice (Fig 5C), the changes in hepatic FAs, metabolite signals, and phospholipids were equalized to WT levels with *long* term fasting. It is also notable that WT and L-KO mice had similar fasting mediated increases in hepatic TGs (Fig 5A) and long-chain FAs (Table 2).

### Liver AMPK deletion increases BCAA/BCKA-related metabolites

Acylcarnitines form from their acyl-CoA species [47]. Catabolism of BCAA/BCKAs yields oxidative (acetyl-CoA) and anaplerotic (succinyl-CoA) substrates [48]. Fasting resulted in an increase in several BCAA/BCKA-related metabolites in WT mice (Table 3). Leucine degradation yields the intermediates isovaleryl-CoA and 3-methylglutaryl-CoA. Metabolites formed from these molecules (beta-hydroxyisovalerate and 3-methylglutarylcarnitine) were elevated in the livers of *long* term fasted livers of WT mice (Table 3). Isobutyryl-CoA and 3-hydroxybutyryl-CoA are intermediates in the catabolism of valine and certain FAs. Their carnitine conjugates (isobutyrylcarnitine and hydroxybutyrylcarnitine, respectively) were also elevated in the livers of *long* term fasted WT mice (Table 3). *Long* term fasting also increased liver acetylcarnitine and glutarylcarnitine, a metabolite associated with lysine catabolism, in WT mice.

**Table 3:** AMPK deletion results in aberrant BCAA/BCKA-related metabolism.

	Effect of Fasting		Effect of Genotype	
*BCAA-Related Metabolites*	WT Long WT Short	L-KO Long L-KO Short	L-KO Short WT Short	L-KO Long WT Long
glutarylcarnitine (C5)	1.34*	1.07	1.35*	1.08
beta-hydroxyisovalerate	1.55*	1.21	1.43*	1.12
4-methyl-2-oxopentanoate	1.07	1.05	1.4*	1.37
alpha-hydroxyisovalerate	1.94*	1.97*	1.24	1.26
isobutyrylcarnitine	2.31*	1.2	2.55*	1.33
2-methylbutyrylcarnitine (C5)	1.48	1.49	1.84	1.85*
3-methylglutarylcarnitine (C6)	4.33*	2.63*	2.39*	1.45*
propionylcarnitine	1.12	2.06*	0.88	1.62*
hydroxybutyrylcarnitine	5.41*	6.2*	0.65*	0.74
acetylcarnitine	4.1*	1.66*	1.59	0.64

Liver BCAA/BCKA-related metabolites were determined for *short* and *long* term fasted WT and L-KO mice through metabolomics analysis. Data are expressed as ratios, *n = 7–8* in each group.

*represents *p*≤0.05 between identified groups.

Fasting also caused a similar effect in livers of L-KO mice. Specifically, alpha-hydroxyisovalerate, 3-methylglutarylcarnitine, propionylcarnitine, hydroxybutyrylcarnitine, and acetylcarnitine increased with fasting in L-KO livers (Table 3). Relative to WT mice, some BCAA/BCKA-related metabolites were increased in the livers of *short* term fasted L-KO mice (glutarylcarnitine, beta-hydroxyisovalerate, 4-methyl-2-oxopentanoate, isobutarylcarnitine) and did not change with fast extension (Table 3). However, liver AMPK deletion resulted in an increase in hepatic 2-methylbutyrylcarnitine, 3-methylglutarylcarnitine, and propionylcarnitine in *long* term fasting (Table 3). No fasting or genotype effects were observed for leucine, isoleucine, and valine (data not shown).

## Discussion

Characterizing the fundamental role of AMPK in different physiological states has proven to be a difficult task due to the enzyme’s numerous downstream targets. Genetic models that inhibit or activate hepatic AMPK have provided valuable insight into the enzyme’s function. Pharmacological studies *in vivo* and *in vitro* have enhanced our understanding of the AMPK-dependent and -independent effects of biguanides [19, 49] and AICAR [17, 19, 49]. Understanding hepatic intermediary fluxes is central to understanding genetic and nutritional regulation of liver metabolism [23–26, 37]. Recently developed *in vivo* flux methods [29] were applied here in combination with metabolomics to investigate hepatic AMPK action in distinct hepatic energy states created by different fast durations. These studies demonstrate that hepatic AMPK works to resist the fasting-mediated decline in energy state and associated changes in glucose flux. In the absence of both AMPKα1 and α2 catalytic subunits, prolonged fasting causes elevated CAC-flux, aberrant BCAA/BCKA-related metabolism, and larger deficits in ATP in the liver.

These studies refine our understanding AMPK’s role in glucoregulation through the use of MFA and metabolomics. The association between increased AMPK activation and the ability of biguanides [19, 49–51] and AICAR [17, 19] to reduce blood glucose or inhibit glucose production suggests an overlap in function. Genetic deletion of hepatic LKB1—AMPK’s major covalent activator—results in marked hyperglycemia [50]. Inhibition of other targets of LKB1 phosphorylation, the salt-inducible kinases, also increases glucose production from hepatocytes [52]. Moreover, the LKB1/AMPK pathway negatively regulates mediators of gluconeogenic gene expression [14, 15, 52–54]. Acute control of glucose production [17, 19, 49] and gluconeogenic gene expression [19] by pharmacological activators, however, may occur through AMPK-independent mechanisms. The studies presented here tested the impact of liver AMPK deletion on glucose production from glycogen and gluconeogenesis in moderate and prolonged fasting *in vivo*. Flux from oxaloacetate to PEP (***V***_***PCK***_) and gluconeogenesis from PEP (***V***_***Enol***_) were not different from WT mice in either condition. Thus, hepatic AMPK is not required for normal gluconeogenic flux from the CAC in two important physiological states.

A decrease in hepatic energy state [1–3] and AMPK activation [2] correspond to endocrine states characterized by increased glucagon action, substrate oxidation, and sustained gluconeogenesis [55]. These observations are consistent with a role for AMPK in oxidative metabolism [5–7]. Recent research has demonstrated that hepatic AMPK deletion impairs respiration [17, 56], paralleling limitations in mitochondrial oxidative metabolism observed in AMPK-deficient muscle [57, 58]. Furthermore, disruption of AMPK’s phosphorylation sites on ACC results in elevated liver malonyl-CoA, hepatocyte lipogenesis, and impaired palmitate oxidation [6]. The studies presented here identify a novel, physiological role for hepatic AMPK in maintaining a metabolic state in the liver that coordinates energy and glucose production.

Indeed, glucose fluxes in *short* term fasted L-KO mice more closely resemble those of a prolonged fast in WT mice. The reduction in glycogenolytic glucose production in *short* term fasted L-KO mice is consistent with reductions in liver glycogen observed following AICAR administration in mice lacking liver AMPK [17] and mice with a whole-body deletion of the AMPKβ2 subunit [59]. AMPK has been shown to phosphorylate and inactivate the predominant isoform of glycogen synthase in the liver [60], suggesting AMPK removal from the liver might increase, rather than decrease, glycogen deposition. However, there are several plausible explanations for the apparent inconsistency between predicted and observed changes in glycogen metabolism. For example, inefficiencies in central oxidative metabolism induced by liver AMPK deletion may necessitate a greater reliance on glycolytic ATP production, thereby reducing G6P available for glycogen synthesis in the post-prandial state. A recent investigation has demonstrated that constitutive liver mTORC1 activation reduces liver glycogen content and glycogenolysis [61]. Thus, genetic removal of AMPK, a negative regulator of mTORC1 signaling, may enable the effects of constitutive mTORC1 signaling on glycogen metabolism. AMPK deficient hepatocytes exhibit reductions in glucokinase expression and glucose phosphorylation [62]. This might also explain reduced liver glycogen.

Mice with a genetic PGC1α deletion [26] or induction of liver hypoxia signaling [63] cause severe impairments in liver metabolism. Both mouse models further substantiate the integration of hepatic oxidative metabolism and liver glucose production. Mice lacking hepatic AMPK exhibit impairments in liver mitochondrial function [17] and a distinct glucose and oxidative phenotype *in vivo*. Glucose production from glycogen is lower in *short* term fasting, yet gluconeogenic flux from the CAC remains intact in L-KO mice. A spectrum of long-chain FAs increase with *short* term fasting, yet liver-AMPK deletion does not cause an abnormal increase in triglycerides in response to a *short* or *long* term fast. Moreover, circulating FAs and triglycerides are no different between 5hr fasted liver AMPK knockout and littermate controls [17]. It is notable that the absence of liver AMPK causes no further effect on glucose fluxes with *long* term fasting. However, *short* term fasted L-KO mice exhibit several metabolic characteristics of livers from *long* term fasted mice. Liver AMPK deletion promotes an early change in phospholipids, the total adenine nucleotide pool, BCAA/BCKA related metabolites, and the aforementioned changes in glucose fluxes and FAs. The majority of these genotype effects are abolished by extended fast duration. This demonstrates that the nutrient stress of a long term fast is drastic enough to either mask or bypass AMPK control of hepatic metabolism.

Acetyl-CoA generation with prolonged fasting appears to exceed its use in the CAC. Accordingly a large proportion of acetyl-CoA derived from fatty-acid β-oxidation is utilized in ketogenesis [37, 64]. Despite a surplus in substrate for oxidation, *long* term fasting in the mouse reduces hepatic ATP and increases the AMP/ATP ratio. Without AMPK, liver ATP levels are significantly lower but, paradoxically, ***V***_***CS***_ is higher than in *long* term fasted WT mice. It is plausible that the elevation in CAC activity is compensatory for an impaired ability to generate reducing equivalents through β-oxidation. Likewise, electron deposition in inefficient electron transport chains [17] may stimulate a greater demand for CAC-derived NADH, FADH_2_, and ATP. A study designed specifically to measure the effect of AMPK deletion on hepatic redox state, ketogenesis and carnitine bound long, medium, and short acyl-CoA species would be helpful in elucidating this paradox. Liver metabolic fluxes have been measured in conditions with an imbalance in substrate availability and CAC activity. The present study examined AMPK control of hepatic energy metabolism and glucose production during nutrient deprivation. Conversely chronic overnutrition has been shown to accelerate *in vivo* CAC flux in the context of inefficient mitochondrial respiration [37]. Furthermore, liver pathologies associated with overnutrition exhibit impairments in hepatic ATP homeostasis [65, 66] and the ketogenic response to fasting [37].

An increase in liver BCAA/BCKA-related metabolites may be further evidence of the disruption in oxidative metabolism caused by AMPK deletion. Indeed, elevations in short-chain acylcarnitine species emerge in other conditions with impaired oxidative metabolism [37, 63]. However, metabolite measurements presented here do not provide sufficient information to assess rates of consumption and production. Interpretation is further limited by interorgan metabolite crosstalk, as noted elsewhere [67]. Flux analysis of hepatic BCAA/BCKA-related metabolism in the presence and absence of AMPK would add insight into the observed phenotype.

Energy status has been a long proposed regulator of metabolism [68–70] and AMPK has received considerable attention for its responsiveness to energy status and the diverse functionality of downstream targets. The disruption of mitochondrial energy metabolism caused by hepatic AMPK removal [17, 56] may act as a primary driver of the impairment in liver oxidative metabolism observed herein. These studies demonstrate hepatic AMPK is a vital component of a molecular signaling network that sustains energy producing pathways during a macronutrient deficit. In the absence of this key enzyme, substrate abundance, oxidation, and ATP production are uncoupled which results in a larger hepatic energy deficit in *long* term fasting *in vivo*.

## Supporting Information

S1 Table Absolute flux estimates (μmol∙kg^-1^·min^-1^) in short and long term fasting and relative flux contribution to V_EndoRa_ in short and long term fasting. Data are average (Ave) and standard error of the mean (SEM).
(PDF)

S2 Table Data for hepatic adenine nucleotides (μmol∙g^-1^) in short and long term fasting and data for liver AMPK and Akt Signaling (A.U.) in short and long term fasting. Data are average (Ave) and standard error of the mean (SEM).
(PDF)

S3 Table Data for liver lipids (μg∙mgLiver-1) in short and long term fasting. Data are average (Ave) and standard error of the mean (SEM).
(PDF)

## Abbreviations

AcCoA: Acetyl-CoA
ACC: Acetyl-CoA carboxylase
Akg: α-Ketoglutarate
AMPK: AMP-activated protein kinase
BPG: 1, 3-Bisphosphoglycerate
BCAA/KA: Branched-chain amino/keto acid
CAC: Citric acid cycle
Cit: Citrate
DHAP: Dihydroxyacetone phosphate
F6P: Fructose-6-phosphate
FBP: Fructose-1, 6-bisphosphate
G6P: Glucose-6-phosphate
GA3P/GAP: Glyceraldehyde-3-phosphate
Lac: Lactate
MFA: Metabolic flux analysis
MID: Mass isotopomer distribution
Oac: Oxaloacetate
PEP: Phosphoenolpyruvate
PropCoA: Propionyl-CoA
Pyr: Pyruvate
SucCoA: Succinyl-CoA
*V_CS_*: Flux from Oac and AcCoA to Cit
*V_EndoRa_*: Endogenous glucose production
*V_Enol_*: Flux from PEP to BPG
*V_GK_*: Flux from glycerol to DHAP
*V_LDH_*: Non-PEP derived, unlabeled sources of anaplerosis to Pyr
*V_PC_*: Flux from Pyr to Oac
*V_PCC_*: Flux from PropCoA to SucCoA
*V_PCK_*: Flux from Oac to PEP
*V_PK+ME_*: Contribution of pyruvate kinase (PK) and malic enzyme (ME) to Pyr
*V_PYGL_*: Flux from glycogen to G6P
*V_SDH_*: Flux from SucCoA to Oac
