# Correction: Liver AMP-Activated Protein Kinase Is Unnecessary for Gluconeogenesis but Protects Energy State during Nutrient Deprivation

**DOI:** 10.1371/journal.pone.0183601

**Published:** 2017-08-16

**Authors:** Clinton M. Hasenour, Martha L. Wall, D. Emerson Ridley, Freyja D. James, Curtis C. Hughey, E. Patrick Donahue, Benoit Viollet, Marc Foretz, Jamey D. Young, David H. Wasserman

Martha L. Wall is not included in the author byline. She should be listed as the second author and is affiliated with the Department of Chemical and Biomolecular Engineering, Vanderbilt University, Nashville, Tennessee, United States of America. The contributions of this author are as follows: Analyzed the data.

Please view the correct author byline, affiliations, and citation here:

Clinton M. Hasenour^1^, Martha L. Wall^2^, D. Emerson Ridley^1^, Freyja D. James^3^, Curtis C. Hughey^1^, E. Patrick Donahue^3^, Benoit Viollet^4,5,6^, Marc Foretz^4,5,6^, Jamey D. Young^1,2^, David H. Wasserman^1,3^

**1** Department of Molecular Physiology and Biophysics, Vanderbilt University, Nashville, Tennessee, United States of America, **2** Department of Chemical and Biomolecular Engineering, Vanderbilt University, Nashville, Tennessee, United States of America, **3** Mouse Metabolic Phenotyping Center, Vanderbilt University, Nashville, Tennessee, United States of America, **4** Inserm, U1016, Institut Cochin, Paris, France, **5** CNRS, UMR 8104, Paris, France, **6** Université Paris Descartes, Sorborne Paris Cité, Paris, France

Hasenour CM, Wall ML, Ridley DE, James FD, Hughey CC, Donahue EP, et al. (2017) Liver AMP-Activated Protein Kinase Is Unnecessary for Gluconeogenesis but Protects Energy State during Nutrient Deprivation. PLoS ONE 12(1): e0170382. https://doi.org/10.1371/journal.pone.0170382
