# Supplementary material for: Liver AMP-Activated Protein Kinase Is Unnecessary for Gluconeogenesis but Protects Energy State during Nutrient Deprivation
Source: PLoS One. 2017 Jan 20;12(1):e0170382. doi: 10.1371/journal.pone.0170382 (PMC5249187; doi:10.1371/journal.pone.0170382)
Supplement: S1 Table — Data are average (Ave) and standard error of the mean (SEM). (PDF) [file pone.0170382.s001.pdf]

S1 Tables

Absolute flux estimates (µmol·kg^{-1}·min^{-1}) in *short* and *long* term fasting

	V_{EndoRa}				V_{PYGL}				V_{GK}			
	**Short**		**Long**		**Short**		**Long**		**Short**		**Long**	
	**WT**	**L-KO**	**WT**	**L-KO**	**WT**	**L-KO**	**WT**	**L-KO**	**WT**	**L-KO**	**WT**	**L-KO**
**Ave**	85.9	73.8	73.3	77.4	27.6	14.1	2.1	1.7	6.2	11.1	19.5	19.2
**SEM**	2.6	3.6	5.2	4.9	2.8	5.3	1.4	1.1	2.2	3.1	2.3	1.7

	V_{Enol}				V_{PCK}				V_{PC}			
	**Short**		**Long**		**Short**		**Long**		**Short**		**Long**	
	**WT**	**L-KO**	**WT**	**L-KO**	**WT**	**L-KO**	**WT**	**L-KO**	**WT**	**L-KO**	**WT**	**L-KO**
**Ave**	52.1	48.6	51.8	56.5	212.6	194.9	229.6	245.1	190.4	172.3	203.8	218.0
**SEM**	1.2	1.7	3.8	4.4	14.1	16.6	26.9	21.3	13.5	15.4	25.7	19.8

	V_{LDH}				V_{PK+ME}				V_{CS}			
	**Short**		**Long**		**Short**		**Long**		**Short**		**Long**	
	**WT**	**L-KO**	**WT**	**L-KO**	**WT**	**L-KO**	**WT**	**L-KO**	**WT**	**L-KO**	**WT**	**L-KO**
**Ave**	82.0	74.7	77.7	85.9	108.4	97.6	126.0	132.1	85.6	79.7	72.7	100.7
**SEM**	2.5	2.7	6.4	7.1	12.7	13.7	19.5	13.9	5.0	5.1	7.9	7.9

	V_{SDH}				V_{PCC}			
	**Short**		**Long**		**Short**		**Long**	
	**WT**	**L-KO**	**WT**	**L-KO**	**WT**	**L-KO**	**WT**	**L-KO**
**Ave**	107.9	102.2	98.5	127.8	22.2	22.5	25.9	27.1
**SEM**	5.3	5.8	9.2	8.7	0.8	1.5	1.4	1.8

Relative flux contribution to V_{EndoRa} in *short* and *long* term fasting

	V_{PYGL}/V_{EndoRa}				V_{GK}/V_{EndoRa}				V_{Enol}/V_{EndoRa}			
	**Short**		**Long**		**Short**		**Long**		**Short**		**Long**	
	**WT**	**L-KO**	**WT**	**L-KO**	**WT**	**L-KO**	**WT**	**L-KO**	**WT**	**L-KO**	**WT**	**L-KO**
**Ave**	0.32	0.18	0.03	0.02	0.07	0.16	0.27	0.25	0.61	0.67	0.71	0.73
**SEM**	0.03	0.06	0.02	0.02	0.02	0.04	0.03	0.02	0.01	0.03	0.01	0.01
