# Supplementary material for: Liver AMP-Activated Protein Kinase Is Unnecessary for Gluconeogenesis but Protects Energy State during Nutrient Deprivation
Source: PLoS One. 2017 Jan 20;12(1):e0170382. doi: 10.1371/journal.pone.0170382 (PMC5249187; doi:10.1371/journal.pone.0170382)
Supplement: S2 Table — Data are average (Ave) and standard error of the mean (SEM). (PDF) [file pone.0170382.s002.pdf]

S2 Tables

Data for hepatic adenine nucleotides ($\mu mol \cdot g^{-1}$) in *short* and *long* term fasting

	ATP				ADP				AMP			
	Short		Long		Short		Long		Short		Long	
	WT	L-KO	WT	L-KO	WT	L-KO	WT	L-KO	WT	L-KO	WT	L-KO
**Ave**	2.74	2.60	1.87	1.40	2.09	1.95	2.14	1.80	0.69	0.61	0.92	0.98
**SEM**	0.09	0.14	0.15	0.07	0.08	0.04	0.03	0.03	0.03	0.06	0.09	0.05

	TAN				EC				AMP/ATP			
	Short		Long		Short		Long		Short		Long	
	WT	L-KO	WT	L-KO	WT	L-KO	WT	L-KO	WT	L-KO	WT	L-KO
**Ave**	5.52	5.17	4.92	4.17	0.69	0.69	0.60	0.55	0.25	0.25	0.53	0.72
**SEM**	0.09	0.07	0.09	0.05	0.01	0.02	0.02	0.01	0.02	0.04	0.10	0.07

Data for liver AMPK and Akt Signaling (A.U.) in *short* and *long* term fasting

	$pAMPK^{T172}/AMPK$		$pACC^{S79}/ACC$		$pAkt^{S473}/Akt$			
	WT		WT		Short		Long	
	Short	Long	Short	Long	WT	L-KO	WT	L-KO
**Ave**	1.05	1.21	1.18	1.03	1.23	1.21	0.64	0.54
**SEM**	0.03	0.02	0.11	0.06	0.07	0.05	0.09	0.06
