# Supplementary material for: Liver AMP-Activated Protein Kinase Is Unnecessary for Gluconeogenesis but Protects Energy State during Nutrient Deprivation
Source: PLoS One. 2017 Jan 20;12(1):e0170382. doi: 10.1371/journal.pone.0170382 (PMC5249187; doi:10.1371/journal.pone.0170382)
Supplement: S3 Table — Data are average (Ave) and standard error of the mean (SEM). (PDF) [file pone.0170382.s003.pdf]

S3 Tables

Data for liver lipids (μg·mgLiver^{-1}) in *short* and *long* term fasting

	Liver TGs				Liver DGs			
	Short		Long		Short		Long	
	WT	L-KO	WT	L-KO	WT	L-KO	WT	L-KO
**Ave**	6.2	7.7	56.8	62.5	0.18	0.23	0.49	0.42
**SEM**	0.9	0.7	5.0	7.5	0.02	0.03	0.11	0.03

	Liver CEs				Liver PLs			
	Short		Long		Short		Long	
	WT	L-KO	WT	L-KO	WT	L-KO	WT	L-KO
**Ave**	0.47	0.49	0.81	0.99	37.2	24.1	28.7	26.8
**SEM**	0.03	0.04	0.06	0.05	2.7	1.1	0.4	0.4
